# Diversity-Oriented Synthesis of a Library of Substituted Tetrahydropyrones Using Oxidative Carbon-Hydrogen Bond Activation and Click Chemistry

**DOI:** 10.3390/molecules16053648

**Published:** 2011-05-02

**Authors:** Nilesh Zaware, Matthew G. LaPorte, Ramy Farid, Lei Liu, Peter Wipf, Paul E. Floreancig

**Affiliations:** 1 Department of Chemistry and Center for Chemical Methodologies & Library Development, University of Pittsburgh, Pittsburgh, PA 15260, USA; 2 Schrödinger, Inc., 120 West 45^th^ Street, New York, NY 10036, USA

**Keywords:** Tetrahydropyran, C-H activation, click-chemistry, diversity-oriented synthesis

## Abstract

Eighteen (2*RS*,6*RS*)-2-(4-methoxyphenyl)-6-(substituted ethyl)dihydro-2*H*-pyran-4(3*H*)ones were synthesized via a DDQ-mediated oxidative carbon-hydrogen bond activation reaction. Fourteen of these tetrahydropyrans were substituted with triazoles readily assembled via azide-alkyne click-chemistry reactions. Examples of a linked benzotriazole and pyrazole motif were also prepared. To complement the structural diversity, the alcohol substrates were obtained from stereoselective reductions of the tetrahydropyrone. This library provides rapid access to structurally diverse non-natural compounds to be screened against a variety of biological targets.

## 1. Introduction

Natural products and their derivatives continue to provide innovative sources for drug discovery [1,2,3,4]. However, many naturally occurring substrates appear to selectively target more highly connected networks associated with essential biological pathways [5,6]. The aims of the NIH supported Centers for Methodology and Library Design (CMLDs) are to produce diverse chemical libraries using novel synthetic methodologies [7]. These non-natural compound collections are delivered to High Throughput Screening (HTS) centers for biological evaluation. Novel chemical scaffolds (*i.e.*, chemotypes) can provide opportunities to investigate biological targets or pathways that may be inaccessible using only the array of currently known natural products, clinically used compounds, or traditional (hetero)aromatic building blocks. In this communication, we describe our diversity-oriented synthesis (DOS) approach to generate a structurally diverse set of triazole-substituted tetrahydropyrans [8,9]. The chemical diversity of these compounds was evaluated against a 5 million “drug-like” compound database (*vide infra*). 

**Figure 1:**
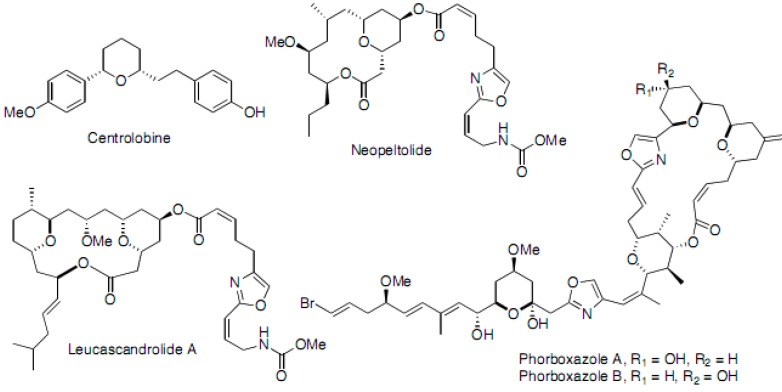
Examples of natural products with a tetrahydropyran core unit.

Tetrahydropyrans are important structural motifs found in a wide array of natural products, from glucose and centrolobine [10,11,12] to structurally elaborate metabolites such as neopeltolide [13,14,15] leucascandrolide A [16] phorboxazole A and B [17,18] and the even more complex architectures present in palytoxin, maitotoxin, and other marine natural products (Figure 1) [19,20].

We have recently demonstrated that tetrahydropyrans (THPs) can be formed in high diastereocontrol from benzylic and allylic ethers through a DDQ-mediated (2,3-dichloro-5,6-dicyano-1,4-benzoquinone) oxocarbenium ion formation followed by an intramolecular nucleophilic addition [21,22]. This versatile methodology is both step [23] and atom [24] efficient, and has been successfully applied to a series of THP-containing natural products [25,26,27,28]. This approach can generate a variety of functionalized THP ring systems and allow for rapid side chain diversification. This report will focus on the construction of triazole- (and related heterocyclic) tethered tetrahydropyrones using azide-alkyne ‘click’ chemistry [29,30]. 

## 2. Results and Discussion

**Scheme 1:**
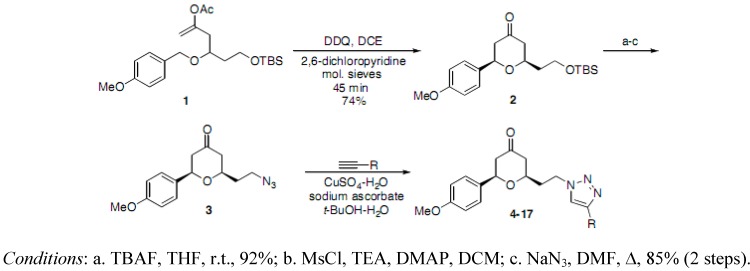
Preparation of triazoles **4-17**.

The *p*-methoxybenzyl (PMB) ether **1** was converted into the substituted tetrahydropyrone **2** using our DDQ-mediated oxidative cyclization protocol [21,22]. The corresponding azide intermediate **3** was synthesized using standard conversions. A stepwise copper (I)-catalyzed Huisgen cycloaddition process was used to couple **3** with various terminal acetylenes to afford the 1,4-disubstituted 1,2,3-triazoles **4-17** (Scheme 1 and Table 1) [29,30]. The alkynes were selected to produce a series of structurally diverse triazoles, all of which gave acceptable calculated physiochemical properties including clogP values [31,32]. Protodesilylation of analog **6** with TBAF and acetic acid furnished the unsubstituted triazole **6** in 29% yield over two steps.

**Table 1:** Tetrazoles **4-16**.

Compound number	R	Yield (%)	clogP
**4**	Ph	71	3.8
**5**	SiMe_3_	54	3.8
**6**	H	29 (two steps)	2.0
**7**	Me	55	2.4
**8**	*^i^*Pr	46	3.2
**9**	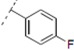	72	4.0
**10**	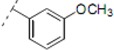	92	3.9
**11**	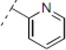	86	3.1
**12**	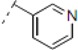	83	2.7
**13**	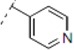	28	2.7
**14**	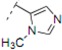	87	2.6
**15**	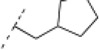	86	4.0
**16**	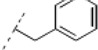	84	4.1
**17**	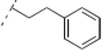	82	4.5

**Scheme 2:**
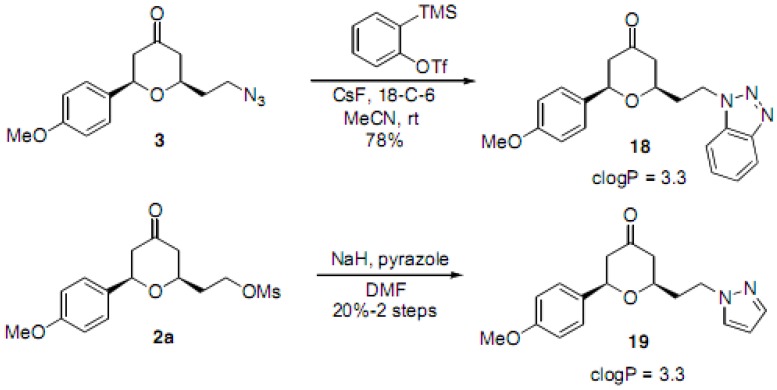
Additional heterocyclic substitutions of the core THP system.

In order to further expand the chemical diversity within this series, a few additional non-triazole containing products were prepared (Scheme 2). The azide intermediate **3** underwent cycloaddition with benzyne (generated *in situ* from *o*-TMS-phenyl triflate) to provide the benzotriazole **18** [33,34]. An additional example involved the synthesis of the pyrazole derivative **19** that was formed from the displacement of the corresponding mesylate in **2a**. 

**Scheme 3:**
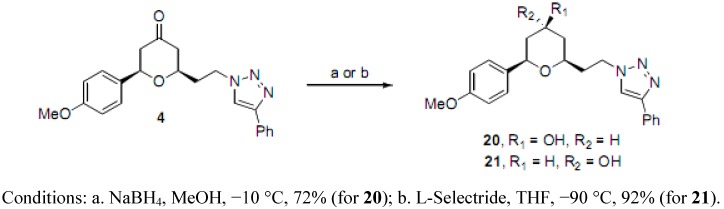
Preparation of alcohols.

A final stereochemical diversity element was incorporated through the preparation of the *syn*- and *anti*-tetrahydropyranols, **20** and **21 **(Scheme 3). We have previously accomplished stereoselective ketone reductions of similar 2,6-disubstituted tetrahydropyrones through the use of L-Selectride or NaBH_4_. [27] Thus, the 4-phenyltriazole **4** was further elaborated to selectively afford the alcohols **20** and **21**. 

**Figure 2:**
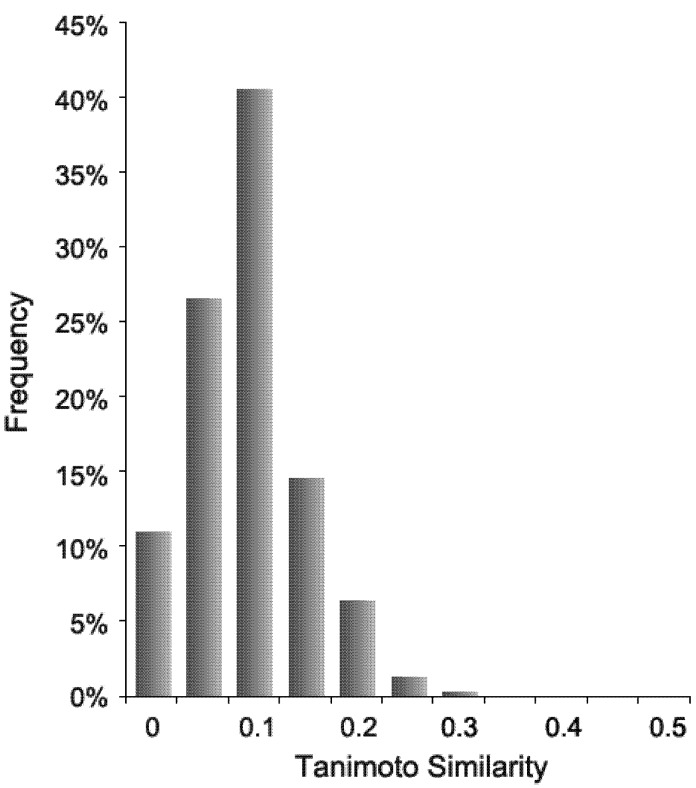
Distribution of similarities between 5 million drug-like, commercially available compounds and the most similar tetrahydropyrans.

With the completion of the triazole and the corresponding heterocycles library, a diversity analysis of the 18 THP products was performed against a set of 5 million commercially available, “drug-like” compounds [35] using the cheminformatics package Canvas [36] and MOLPRINT2D [37,38] hashed binary fingerprints (32-bit, no scaling) with the default Mol2 atom types. This combination of fingerprint and atom type was chosen because it provides the best overall results for virtual screening enrichment across a wide range of targets [39,40]. Using the Tanimoto similarity metric, the tetrahydropyrans described herein were found to be highly diverse compaired to the 5 million drug-like compounds (Figure 2). The average maximum similarity for the 18 products to any member of the 5 million compound database is 0.41, which is remarkably low considering the large number of compounds in the latter repository. The highest similarity is 0.55 (observed for **4**, which along with **18** shows the highest similarity to the 5 million compound set). The least similar compound is **14**, which has a maximum similarity to any one of the 5 million compounds of only 0.27. Figure 2 shows the distribution of similarities between each of the 5 million compounds and the one compound of the 18 THPs that in each case is most similar. The figure shows that a large majority of the 5 million “drug-like” compounds is highly dissimilar to the 18 THPs. 

## 3. Experimental Section

### 3.1. General

All reactions were performed under an argon atmosphere and all glassware was flame dried prior to use. CH_2_Cl_2 _and THF were dried by passing through a column of activated alumina. Reactions carried out at −78 °C employed a CO_2_/acetone bath. Reactions were monitored by TLC analysis (pre-coated silica gel 60 F254 plates, 250 μm layer thickness) and visualization was accomplished with a 254 nm UV light and by staining with a KMnO_4_ solution (1.5 g of KMnO_4_ and 1.5 g of K_2_CO_3_ in 100 mL of a 0.1% NaOH solution), CAM solution (5 g of cerium sulfate, 25 g of ammonium molybdate, 50 mL of conc. H_2_SO_4_ and 450 mL of H_2_O, *p*-anisaldehyde solution (2.5 mL of *p*-anisaldehyde, 2 mL of AcOH, and 3.5 mL of conc. H_2_SO_4_ in 100 mL of 95% EtOH). Purifications by chromatography were performed using SiO_2_ (SiliaFlash® F60, Silicycle). ^1^H-NMR spectra were recorded on Bruker Avance 300/400/600 MHz instruments in CDCl_3_. Chemical shifts were reported in parts per million with the residual solvent peak used as an internal standard. Chemical shifts are tabulated as follows: chemical shift, multiplicity (s = singlet, d = doublet, t = triplet, q = quartet, m = multiplet, bs = broad singlet, dd = doublet of doublet, dt = doublet of triplet, dq = doublet of quartet, sp = septet), coupling constant(s), and integration. ^13^C NMR spectra were run at 75 or 100 MHz using a proton-decoupled pulse sequence with a d1 of 3 sec, and are tabulated by observed peak. Mass spectra were obtained on a Micromass Autospec double focusing instrument. IR spectra were obtained on a Identify IR-ATR spectrometer.

*(2RS,6RS)-2-(2-Hydroxyethyl)-6-(4-methoxyphenyl)dihydro-2H-pyran-4(3H)-one*. To a solution of (2*RS*,6*RS*)-2-(2-((*tert*-butyldimethylsilyl)oxy)ethyl)-6-(4-methoxyphenyl)dihydro-2*H*-pyran-4(3*H*)-one **2 [21]** (0.104 g, 0.285 mmol) in THF (3.5 mL) was added TBAF (0.429 mL, 1 M solution in THF) dropwise at 0 °C over 10 min. The reaction mixture was allowed to warm to r.t. and stirred for 1.5 h, quenched with water (5 mL), extracted with DCM, dried (Na_2_SO_4_), filtered, concentrated under reduced pressure, and purified by chromatography (SiO_2_) eluting with hexane-ethyl acetate (3:7) to afford the primary alcohol (0.065 g, 92%) as a colorless oil: IR (neat) 3419, 2954, 2915, 1711, 1610, 1513, 1461, 1302, 1245, 1174, 1149, 1031, 826 cm^−1^; ^1^H-NMR (300 MHz, CDCl_3_) δ 7.29 (d, *J* = 8.4 Hz, 2H), 6.92 (d, *J =* 8.7 Hz, 2H), 4.65 (t, *J* = 7.2 Hz, 1H), 4.07–4.03 (m, 1H), 3.86 (q, *J* = 6.0 Hz, 2H), 3.82 (s, 3H), 2.61 (d, *J* = 7.8 Hz, 2H), 2.48 (d, *J* = 7.8 Hz, 2H), 2.21 (t, *J* = 5.1 Hz, 1H), 2.07–1.97 (m, 1H), 1.95–1.85 (m, 1H); ^13^C-NMR (75 MHz, CDCl_3_) δ 206.5, 159.7, 132.8, 127.2, 114.3, 78.9, 60.5, 55.6, 55.5, 49.6, 47.9, 38.7; HRMS (ESI) *m/z* calcd for C_14_H_18_O_4_Na (M+Na) 273.1103, found 273.1118. 

*(2RS,6RS)-2-(2-Azidoethyl)-6-(4-methoxyphenyl)dihydro-2H-pyran-4(3H)-one* (**3**). To a solution of the alcohol intermediate (0.122 g, 0.487 mmol), triethylamine (0.103 mL, 0.731 mmol) and DMAP (0.003 g, 0.025 mmol) in DCM (2.5 mL) was added methanesulfonyl chloride (0.045 mL, 0.576 mmol) dropwise at 0 °C. The resulting solution was stirred at r.t. for 15 min. The reaction mixture was diluted with HCl (0.5 M) and extracted with DCM. The aqueous layer was back extracted with DCM. The combined organic layers were washed with saturated NaHCO_3_, brine, dried (Na_2_SO_4_), filtered and concentrated under reduced pressure to obtain the crude 2-((2*RS*,6*RS*)-6-(4-methoxyphenyl)-4-oxotetrahydro-2*H*-pyran-2-yl)ethyl methanesulfonate **2a **(0.171 g) as a yellow oil which was used without further purification: ^1^H-NMR (400 MHz, CDCl_3_) δ 7.29 (d, *J =* 8.4 Hz, 2H), 6.92 (d, *J =* 8.4 Hz, 2H,), 4.63 (dd, *J =* 4.4, 9.6 Hz, 1H), 4.51–4.45 (m, 1H), 4.42–4.38 (m, 1H), 4.01–3.95 (m, 1H), 3.83 (s, 3H), 2.98 (s, 3H), 2.63-2.59 (m, 2H), 2.52-2.38 (m, 2H), 2.12-2.07 (m, 2H). To a solution of **2a** (0.171 g, 0.520 mmol) in DMF (1 mL) was added sodium azide (0.063 g, 0.969 mmol) at r.t. The reaction mixture was stirred at r.t. for 13.5 h. TLC analysis indicated remaining starting material. The reaction mixture was then heated at 40 °C for 6 h and at 50 °C for 1 h, diluted with water, extracted with diethyl ether, dried (Na_2_SO_4_), filtered, concentrated under reduced pressure and purified by chromatography on SiO_2_ (hexane/EtOAc, 3:7) to afford the azide **3** (0.114 g, 85% over two steps) as a colorless oil: IR (neat) 3424, 2098, 1717, 1614, 1515, 1461, 1355, 1303, 1247, 1177, 1032 cm^−1^; ^1^H-NMR (300 MHz, CDCl_3_) δ 7.29 (d, *J =* 8.7 Hz, 2H), 6.92 (d, *J =* 8.7 Hz, 2H), 4.62 (dd, *J =* 4.8, 9.6 Hz, 1H), 3.94–3.86 (m, 1H), 3.81 (s, 3H), 3.55–3.46 (m, 2H), 2.65–2.58 (m, 2H), 2.50–2.35 (m, 2H), 1.99–1.85 (m, 2H); ^13^C-NMR (100 MHz, CDCl_3_) δ 206.2, 159.5, 132.7, 127.1, 114.0, 78.3, 74.1, 55.3, 49.2, 47.6, 47.5, 35.6; MS (EI) *m/z* 275 (M+, 25%), 135 (100); HRMS (EI) *m/z* calcd for C_14_H_17_N_3_O_3_ 275.1269, found 275.1265. 

*(2RS,6RS)-2-(4-Methoxyphenyl)-6-(2-(4-phenyl-1H-1,2,3-triazol-1-yl)ethyl)dihydro-2H-pyran-4(3H)-one* (**4**). To a suspension of **3** (0.114 g, 0.414 mmol) and phenylacetylene (0.045 mL, 0.410 mmol) in a mixture of water and *tert*-butyl alcohol (1:1, 1.7 mL) was added sodium ascorbate (0.032 mL, 1 M solution in water), followed by copper(II) sulfate pentahydrate (0.001 g, 0.004 mmol). The heterogeneous mixture was stirred at r.t. for 33 h, recharged with phenylacetylene (0.090 mL, 0.820 mmol) and stirred at r.t. for 21 h. The reaction mixture was diluted with water (7 mL), cooled in ice bath, and filtered. The precipitate was dissolved in ethyl acetate and chloroform, dried (Na_2_SO_4_), filtered, and concentrated to afford the corresponding triazole **4 **(0.110 g, 71%) as an off-white solid: m.p. 151–152 °C; IR (neat) 3089, 2964, 1714, 1611, 1515, 1470, 1355, 1242, 1174, 1036, 816, 769 cm^−1^; ^1^H-NMR (300 MHz, CDCl_3_) δ 7.77 (d, *J =* 6.9 Hz, 2H), 7.72 (s, 1H), 7.42 (t, *J* = 6.9 Hz, 2H), 7.35–7.32 (m, 1H), 7.29 (d, *J =* 8.7 Hz, 2H), 6.91 (d, *J =* 8.7 Hz, 2H), 4.62–4.54 (m, 3H), 3.81 (s, 3H), 3.79–3.72 (m, 1H), 2.59 (d, *J =* 7.5 Hz, 2H), 2.45–2.42 (m, 2H), 2.32–2.25 (m, 2H); ^13^C-NMR (75 MHz, CDCl_3_) δ 205.7, 159.7, 147.8, 132.6, 130.7, 128.9, 128.3, 127.3, 125.8, 120.3, 114.3, 78.7, 74.2, 55.5, 49.3, 47.4, 46.9, 36.6; HRMS (ESI) *m/z* calcd for C_22_H_23_N_3_O_3_Na (M+Na) 400.1637, found 400.1642. 

*(2RS,6RS)-2-(4-Methoxyphenyl)-6-(2-(4-(trimethylsilyl)-1H-1,2,3-triazol-1-yl)ethyl)dihydro-2H-pyran-4(3H)-one* (**5**). To a suspension of **3** (0.072 g, 0.262 mmol) and trimethylsilylacetylene (0.075 mL, 0.529 mmol) in a mixture of water and *tert*-butyl alcohol (1:1, 1.1 mL) was added sodium ascorbate (0.025 mL, 1 M solution in water) followed by copper(II) sulfate pentahydrate (0.001 g, 0.004 mmol). The heterogeneous mixture was stirred at r.t. for 14 h, recharged with trimethylsilylacetylene (0.075 mL, 0.529 mmol) and stirred at r.t. for an additional 6 h. The reaction mixture was diluted with water, extracted with diethyl ether, dried (Na_2_SO_4_), filtered, concentrated and purified by chromatography (SiO_2_) eluting with hexane-ethyl acetate (1:1) to obtain the triazole **5** (0.053 g, 54%) as a colorless oil: IR (neat) 2958, 1717, 1614, 1515, 1463, 1351, 1248, 1177, 1035, 839, 752 cm^−1^; ^1^H-NMR (300 MHz, CDCl_3_) δ 7.46 (s, 1H), 7.31 (d, *J* = 8.7 Hz, 2H), 6.93 (d, *J =* 8.7 Hz, 2H), 4.61–4.51 (m, 3H), 3.83 (s, 3H), 3.75–3.65 (m, 1H), 2.61–2.58 (d, *J=* 7.2 Hz, 2H), 2.43–2.39 (m, 2H), 2.32–2.19 (m, 2H), 0.30 (s, 9H); ^13^C-NMR (75 MHz, CDCl_3_) δ 205.9, 159.8, 146.7, 132.7, 129.5, 127.3, 114.3, 78.7, 74.0, 55.5, 49.4, 47.5, 46.1, 36.8, −0.9; HRMS (ESI) *m/z* calcd for C_19_H_27_N_3_O_3_Si Na (M+Na) 396.1719, found 396.1698. 

*(2RS,6RS)-2-(2-(1H-1,2,3-Triazol-1-yl)ethyl)-6-(4-methoxyphenyl)dihydro-2H-pyran-4(3H)-one* (**6**). To a solution of **5** (0.050 g, 0.134 mmol) in THF (1.5 mL) was added TBAF (0.402 ml, 1 M solution in THF) dropwise at 0 °C followed by the addition of acetic acid (0.008 mL, 0.140 mmol). The reaction mixture was stirred at r.t. for 14 h, recharged with acetic acid (0.016 mL, 0.280 mmol) and stirred at r.t. for 4 h, concentrated, and purified by chromatography (SiO_2_) eluting with hexane-ethyl acetate (3:7) to afford **6** (0.021 g, 53%) as a colorless oil. IR (neat) 2958, 1713, 1611, 1585, 1514, 1446, 1303, 1247, 1176, 1029, 813 cm^−1^; ^1^H-NMR (400 MHz, CDCl_3_) δ 7.68 (s, 1H), 7.51 (s, 1H), 7.30 (d, *J* = 8.8 Hz, 2H), 6.94 (d, *J =* 8.8 Hz, 2H), 4.62–4.54 (m, 3H), 3.83 (s, 3H), 3.72–3.65 (m, 1H), 2.61 (d, *J=* 8.8 Hz, 2H), 2.45–2.37 (m, 2H), 2.31–2.21 (m, 2H); ^13^C-NMR (100 MHz, CDCl_3_) δ 205.7, 159.8, 133.9, 132.6, 127.3, 123.9, 114.3, 78.7, 73.9, 55.5, 49.4, 47.4, 46.6, 36.7, 29.9; HRMS (ESI) *m/z* calcd for C_16_H_20_N_3_O_3_ (M+H^+^) 302.1505, found 302.1531.

*(2RS,6RS)-2-(4-Methoxyphenyl)-6-(2-(4-methyl-1H-1,2,3-triazol-1-yl)ethyl)dihydro-2H-pyran-4(3H)-one* (**7**). Propyne (1 mL, 17.7 mmol) was condensed into a 10 mL flask using a dry-ice/acetone condenser. A suspension of **3** (0.060 g, 0.218 mmol) in a mixture of water and *tert*-butyl alcohol (1:1, 1.7 mL) was added to the flask, followed by the addition of sodium ascorbate (0.02 mL, 1 M solution in water), and copper(II) sulfate pentahydrate (0.001 g, 0.004 mmol). The reaction mixture was stirred at r.t. for 19 h, diluted with water, extracted with ethyl acetate, dried (Na_2_SO_4_), filtered, concentrated and purified by chromatography (SiO_2_) eluting with hexane-ethyl acetate (2:3) to obtain the triazole **7 **(0.038 g, 55%) as a colorless oil: IR (neat) 3584, 2956, 1715, 1612, 1514, 1303, 1247, 1176, 1049, 1029, 813 cm^−1^; ^1^H-NMR (300 MHz, CDCl_3_) δ 7.29 (d, *J* = 8.7 Hz, 2H), 7.22 (s, 1H), 6.93 (d, *J =* 8.7 Hz, 2H), 4.59–4.49 (m, 3H), 3.83 (s, 3H), 3.74–3.66 (m, 1H), 2.60–2.57 (m, 2H), 2.42–2.39 (m, 2H), 2.32 (s, 3H), 2.27–2.22 (m, 2H); ^13^C-NMR (100 MHz, CDCl_3_) δ 205.9, 159.7, 143.5, 132.7, 127.3, 121.7, 114.2, 78.6, 73.9, 55.5, 49.4, 47.4, 46.5, 36.8, 10.9; MS (EI) *m/z* 338 (M+Na, 100%), 316 (M+H, 18), 298 (18), 233 (50), 215 (75); HRMS (ESI) *m/z* calcd for C_17_H_21_N_3_O_3_Na (M+Na) 338.1481, found 338.1487. 

*(2RS,6RS)-2-(2-(4-Isopropyl-1H-1,2,3-triazol-1-yl)ethyl)-6-(4-methoxyphenyl)dihydro-2H-pyran-4(3H)-one* (**8**). To a suspension of **3** (0.088 g, 0.319 mmol) and 3-methyl-1-butyne (0.065 mL, 0.635 mmol) in a mixture of water and *tert*-butyl alcohol (1:1, 1.1 mL) was added sodium ascorbate (0.03 mL, 1 M solution in water), followed by copper(II) sulfate pentahydrate (0.001 g, 0.004 mmol). The heterogeneous mixture was stirred at r.t. for 18 h, diluted with water, extracted with ethyl acetate, dried (Na_2_SO_4_), filtered, concentrated and purified by chromatography (SiO_2_) eluting with hexane-ethyl acetate (1:1) to obtain the triazole **8** (0.050 g, 46%) as an off-white solid: m.p. 66–67 °C; IR (neat) 3629, 2960, 1715, 1514, 1446, 1245, 1176, 1045, 813 cm^-1^; ^1^H-NMR (300 MHz, CDCl_3_) δ 7.30 (d, *J* = 8.7 Hz, 2H), 7.21 (s, 1H), 6.93 (d, *J =* 8.7 Hz, 2H), 4.58–4.49 (m, 3H), 3.82 (s, 3H), 3.76–3.65 (m, 1H), 3.06 (sp, *J =* 6.9 Hz, 1H), 2.59 (d, *J=* 7.5 Hz, 2H), 2.42–2.39 (m, 2H), 2.28–2.19 (m, 2H), 1.27 (dd, *J =* 1.8, 6.9 Hz, 6H); ^13^C-NMR (75 MHz, CDCl_3_) δ 205.9, 159.7, 154.7, 132.7, 127.3, 119.8, 114.2, 78.7, 74.0, 55.5, 49.4, 47.4, 46.5, 36.7, 25.9, 22.7; MS (EI) *m/z* 366 (M+Na, 100%), 344 (M+H, 55), 326 (40), 233 (50), 215 (60); HRMS (ESI) *m/z* calcd for C_19_H_26_N_3_O_3 _344.1974 (M+H), found 344.1960. 

*(2RS,6RS)-2-(2-(4-(4-Fluorophenyl)-1H-1,2,3-triazol-1-yl)ethyl)-6-(4-methoxyphenyl)dihydro-2H-pyran-4(3H)-one* (**9**). To a suspension of **3** (0.052 g, 0.189 mmol) and 1-ethynyl-4-fluorobenzene (0.043 mL, 0.37 mmol) in a mixture of water and *tert*-butyl alcohol (1:1, 1.1 mL) was added sodium ascorbate (0.02 mL, 1 M solution in water), followed by copper(II) sulfate pentahydrate (0.001 g, 0.004 mmol). The heterogeneous mixture was stirred at r.t. for 12 h, diluted with water and filtered. The solid was dissolved in ethyl acetate, dried (Na_2_SO_4_), filtered, concentrated and purified by chromatography (SiO_2_) eluting with hexane-ethyl acetate (1:1) to obtain the triazole **9** (0.054 g, 72%) as a white solid: m.p. 158–159 °C; IR (neat) 3559, 2957, 1715, 1613, 1514, 1497, 1247, 1176, 1027, 1027, 841, 813 cm^−1^; ^1^H-NMR (300 MHz, CDCl_3_) δ 7.74–7.69 (m, 2H), 7.67 (s, 1H), 7.29 (d, *J* = 8.7 Hz, 2H), 7.10 (app t, *J=* 8.4 Hz, 2H), 6.91 (d, *J =* 8.7 Hz, 2H), 4.67–4.54 (m, 3H), 3.82 (s, 3H), 3.79–3.74 (m, 1H), 2.59 (d, *J=* 7.2 Hz, 2H), 2.49–2.42 (m, 2H), 2.34–2.26 (m, 2H); ^13^C-NMR (75 MHz, CDCl_3_) δ 205.7, 164.5, 161.2, 159.8, 147.0, 132.5, 127.6, 127.5, 127.3, 1267.0, 126.9; 120.1, 116.1, 115.8, 114.3, 78.8, 74.3, 55.5, 49.4, 47.4, 47.0, 36.6; HRMS (ESI) *m/z* calcd for C_22_H_22_FN_3_O_3_Na (M+Na) 418.1543, found 418.1570. 

*(2RS,6RS)-2-(4-Methoxyphenyl)-6-(2-(4-(3-methoxyphenyl)-1H-1,2,3-triazol-1-yl)ethyl)dihydro-2H-pyran-4(3H)-one* (**10**). To a suspension of **3** (0.075 g, 0.272 mmol) and 1-ethynyl-3-methoxybenzene (0.069 mL, 0.543 mmol) in a mixture of water and *tert*-butyl alcohol (1:1, 1.5 mL) was added sodium ascorbate (0.025 mL, 1 M solution in water), followed by copper(II) sulfate pentahydrate (0.001 g, 0.004 mmol). The heterogeneous mixture was stirred at r.t. for 12 h, diluted with water, extracted with ethyl acetate, dried (Na_2_SO_4_), filtered, concentrated and purified by chromatography (SiO_2_) eluting with hexane-ethyl acetate (1:1) to obtain the triazole **10** (0.102 g, 92%) as an off-white solid: m.p. 111–112 °C; IR (neat) 3633, 2957, 1713, 1611, 1585, 1245, 1174, 1040, 813 cm^−1^; ^1^H-NMR (300 MHz, CDCl_3_) δ 7.71 (s, 1H), 7.42–7.41 (m, 1H), 7.34–7.26 (m, 4H), 6.90 (d, *J =* 8.7 Hz, 2H), 6.88–6.86 (m, 1H), 4.68–4.53 (m, 3H), 3.86 (s, 3H), 3.81 (s, 3H), 3.77–3.69 (m, 1H), 2.59–2.57 (d, *J =* 7.5 Hz, 2H), 2.47–2.41 (m, 2H), 2.33–2.24 (m, 2H); ^13^C-NMR (75 MHz, CDCl_3_) δ 205.7, 160.2, 159.7, 147.7, 132.5, 1312.0, 130.0, 127.3, 120.5, 118.2, 114.4, 114.2, 110.8, 78.7, 74.1, 55.5, 55.4, 49.3, 47.4, 46.9, 36.6; MS (EI) *m/z* 430 (M+Na, 90%), 408 (M+H, 60), 365 (70), 233 (15), 215 (20); HRMS (ESI)*m/z* calcd for C_23_H_25_N_3_O_4_Na (M+Na) 430.1743, found 430.1727.

*(2RS,6RS)-2-(4-Methoxyphenyl)-6-(2-(4-(pyridin-2-yl)-1H-1,2,3-triazol-1-yl)ethyl)dihydro-2H-pyran-4(3H)-one* (**11**). To a suspension of **3** (0.080 g, 0.291 mmol) and 2-ethynylpyridine (0.294 mL, 2.91 mmol) in a mixture of water and *tert*-butyl alcohol (1:1, 1.6 mL) was added sodium ascorbate (0.029 mL, 1 M solution in water), followed by copper(II) sulfate pentahydrate (0.001 g, 0.004 mmol). The heterogeneous mixture was stirred at r.t. for 42 h, diluted with water, extracted with ethyl acetate, dried (Na_2_SO_4_), filtered, concentrated and purified by chromatography (SiO_2_) eluting with hexane-ethyl acetate (3:7) to obtain the triazole **11** (0.095 g, 86%) as a brown oil: IR (neat) 3286, 2960, 1713, 1605, 1514, 1245, 1174, 1038, 719 cm^−1^; ^1^H-NMR (400 MHz, CDCl_3_) δ 8.56 (app dq, *J =* 0.8, 0.8, 0.8, 4.8 Hz, 1H), 8.14 (app dt, *J =* 1.2, 1.2, 8.0 Hz, 1H), 8.11 (s, 1H), 7.76 (app dt, *J =* 1.6, 7.6, 7.6 Hz, 1H), 7.31 (d, *J* = 8.8 Hz, 2H), 7.23 (ddd, *J =* 1.2, 4.8, 7.6 Hz, 1H), 6.90 (d, *J =* 8.8 Hz, 2H), 4.69–4.54 (m, 3H), 3.81 (s, 1H), 3.79–3.72 (m, 1H), 2.59 (d, *J =* 7.6 Hz, 2H), 2.48–2.41 (m, 2H), 2.32–2.27 (m, 2H); ^13^C-NMR (100 MHz, CDCl_3_) δ 205.8, 159.7, 150.4, 149.6, 148.6, 137.1, 132.6, 127.4, 123.1, 122.6, 120.4, 114.2, 78.6, 73.9, 55.5, 49.3, 47.4, 46.9, 36.7; HRMS (ESI) *m/z* calcd for C_21_H_22_N_4_O_3_Na (M+Na) 401.1590, found 401.1559. 

*(2RS,6RS)-2-(4-Methoxyphenyl)-6-(2-(4-(pyridin-3-yl)-1H-1,2,3-triazol-1-yl)ethyl)dihydro-2H-pyran-4(3H)-one* (**12**). To a suspension of **3** (0.087 g, 0.316 mmol) and 3-ethynylpyridine (0.065 g, 0.630 mmol) in a mixture of water and *tert*-butyl alcohol (1:1, 1.7 mL) was added sodium ascorbate (0.032 mL, 1 M solution in water), followed by copper(II) sulfate pentahydrate (0.001 g, 0.004 mmol). The heterogeneous mixture was stirred at r.t. for 14 h, diluted with water and filtered. The solid was dissolved in ethyl acetate, dried (Na_2_SO_4_), filtered, concentrated and purified by chromatography (SiO_2_) eluting with hexane-ethyl acetate-triethylamine (1:9:0.1) to obtain the triazole **12** (0.099 g, 83%) as a brown solid: m.p. 146–147 °C; IR (neat) 2960, 1708, 1611, 1514, 1446, 1247, 1174, 1049, 1025, 813, 707 cm^−1^; ^1^H-NMR (300 MHz, CDCl_3_) δ 8.92 (dd, *J* = 0.6, 2.0 Hz, 1H), 8.57 (dd, *J* = 1.5, 4.8 Hz, 1H), 8.14 (app dt, *J* = 1.8, 1.8, 8.3 Hz, 1H) , 7.80 (s, 1H), 7.36 (ddd, *J* = 0.6, 4.8, 8.1 Hz, 1H), 7.28 (d, *J* = 8.4 Hz, 2H), 6.90 (d, *J =* 8.7 Hz, 2H), 4.73–4.54 (m, 3H), 3.82 (s, 3H), 3.79–3.73 (m, 1H), 2.59 (d, *J=* 7.5 Hz, 2H), 2.50–2.43 (m, 2H), 2.39–2.25 (m, 2H); ^13^C-NMR (100 MHz, CDCl_3_) δ 205.6, 159.8, 149.4, 147.2, 144.8, 133.1, 132.5, 127.3, 126.9, 123.9, 120.7, 114.3, 78.9, 74.2, 55.5, 49.4, 47.4, 47.2, 36.6; HRMS (ESI) *m/z* calcd for C_21_H_23_N_4_O_3_ (M+H) 379.1770, found 379.1757. 

*(2RS,6RS)-2-(4-Methoxyphenyl)-6-(2-(4-(pyridin-4-yl)-1H-1,2,3-triazol-1-yl)ethyl)dihydro-2H-pyran-4(3H)-one* (**13**). To a suspension of **3** (0.087 g, 0.316 mmol) and 4-ethynylpyridine hydrochloride (0.088 g, 0.630 mmol) in a mixture of water and *tert*-butyl alcohol (1:1, 1.7 mL) was added sodium ascorbate (0.032 mL, 1 M solution in water), followed by copper(II) sulfate pentahydrate (0.001 g, 0.004 mmol). The heterogeneous mixture was stirred at r.t. for 12 h, diluted with water, triethylamine (1 mL) was added, extracted with ethyl acetate, dried (Na_2_SO_4_), filtered, concentrated and purified by chromatography (SiO_2_) eluting with hexane-ethyl acetate-triethylamine (1:9:0.1). The residue was washed with saturated sodium bicarbonate, extracted with ethyl acetate, dried (Na_2_SO_4_), filtered and concentrated to obtain the triazole **13** (0.034 g, 28%) as an off-white solid: m.p. 144–145 °C; IR (neat) 3038, 2959, 1715, 1610, 1247, 1213, 746, 719 cm^−1^; ^1^H-NMR (400 MHz, CDCl_3_) δ 8.65 (bs, 2H), 7.85 (s, 1H), 7.63 (bs, 2H), 7.27 (d, *J* = 8.4 Hz, 2H), 6.90 (d, *J =* 8.8 Hz, 2H), 4.73–4.52 (m, 3H), 3.81 (s, 3H), 3.79–3.74 (m, 1H), 2.59 (d, *J =* 7.6 Hz, 2H), 2.49–2.40 (m, 2H), 2.35–2.28 (m, 2H); ^13^C- NMR (100 MHz, CDCl_3_) δ 205.5, 159.8, 150.6, 145.3, 138.1, 132.4, 127.3, 121.9, 114.3, 78.9, 74.4, 55.5, 49.4, 47.4, 47.3, 36.5, 29.9; HRMS (ESI) *m/z* calcd for C_21_H_23_N_4_O_3_ (M+H) 379.1770, found 379.1754.

*(2RS,6RS)-2-(4-Methoxyphenyl)-6-(2-(4-(1-methyl-1H-imidazol-5-yl)-1H-1,2,3-triazol-1-yl)ethyl)-dihydro-2H-pyran-4(3H)-one* (**14**). To a suspension of **3** (0.075 g, 0.272 mmol) and 5-ethynyl-1-methyl-1*H*-imidazole (0.056 mL, 0.551 mmol) in a mixture of water and *tert*-butyl alcohol (1:1, 1.5 mL) was added sodium ascorbate (0.025 mL, 1 M solution in water), followed by copper(II) sulfate pentahydrate (0.001 g, 0.004 mmol). The heterogeneous mixture was stirred at r.t. for 12 h, diluted with water, extracted with ethyl acetate, dried (Na_2_SO_4_), filtered, concentrated and purified by chromatography (SiO_2_) eluting with chloroform-methanol (20:1) to obtain the triazole **14** (0.090 g, 87%) as a colorless oil: IR (neat) 3003, 1713, 1610, 1514, 1245, 1174, 1027, 747 cm^−1^;^ 1^H-NMR (300 MHz, CDCl_3_) δ 7.63 (s, 1H), 7.48 (bs, 1H), 7.27 (d, *J* = 8.7 Hz, 2H), 7.15 (bs, 1H), 6.89 (d, *J =* 8.7 Hz, 2H), 4.68–4.52 (m, 3H), 3.88 (s, 3H), 3.81 (s, 3H), 3.78–3.67 (m, 1H), 2.58 (d, *J =* 7.2 Hz, 2H), 2.48–2.36 (m, 2H), 2.33–2.22 (m, 2H); ^13^C-NMR (100 MHz, CDCl_3_) δ 205.6, 159.7, 139.6, 138.7, 132.4, 128.6, 127.2, 123.5, 121.5, 114.2, 78.7, 74.0, 55.5, 49.3, 47.4, 46.9, 36.5, 33.8; MS (EI) *m/z* 405 (M+Na, 20%), 382 (M+H, 60), 365 (100); HRMS (ESI) *m/z* calcd for C_20_H_24_N_5_O_3_ (M+H) 382.1879, found 382.1854. 

*(2RS,6RS)-2-(2-(4-(Cyclopentylmethyl)-1H-1,2,3-triazol-1-yl)ethyl)-6-(4-methoxyphenyl)dihydro-2H-pyran-4(3H)-one* (**15**). To a suspension of **3** (0.072 g, 0.262 mmol) and 3-cyclopentyl-1-propyne (0.068 mL, 0.520 mmol) in a mixture of water and *tert*-butyl alcohol (1:1, 1.1 mL) was added sodium ascorbate (0.025 mL, 1 M solution in water), followed by copper(II) sulfate pentahydrate (0.001 g, 0.004 mmol). The heterogeneous mixture was stirred at r.t. for 17 h, diluted with water, extracted with diethyl ether, dried (Na_2_SO_4_), filtered, concentrated and purified by chromatography (SiO_2_) eluting with hexane-ethyl acetate (1:1) to obtain the triazole **15** (0.086 g, 86%) as a colorless oil: IR (neat) 2945, 1715, 1613, 1514, 1446, 1247, 1174, 1045, 828, 813 cm^−1^; ^1^H-NMR (300 MHz, CDCl_3_) δ 7.29 (d, *J* = 8.7 Hz, 2H), 7.22 (s, 1H), 6.92 (d, *J =* 8.7 Hz, 2H), 4.57–4.49 (m, 3H), 3.82 (s, 3H), 3.73–3.64 (m, 1H), 2.59 (d, *J =* 6.0 Hz, 2H), 2.42–2.38 (m, 2H), 2.29–2.06 (m, 3H), 1.78–1.68 (m, 2H), 1.63–1.51 (m, 4H), 1.25–1.17 (m, 2H); ^13^C-NMR (100 MHz, CDCl_3_) δ 205.9, 159.7, 148.0, 132.6, 127.2, 121.4, 114.2, 78.6, 73.9, 55.5, 49.4, 47.4, 46.4, 40.1, 36.7, 32.6, 31.8, 25.2; HRMS (ESI) *m/z* calcd for C_22_H_29_N_3_O_3_Na (M+Na) 406.2107, found 406.2069. 

*(2RS,6RS)-2-(2-(4-Benzyl-1H-1,2,3-triazol-1-yl)ethyl)-6-(4-methoxyphenyl)dihydro-2H-pyran-4(3H)-one* (**16**). To a suspension of **3** (0.072 g, 0.262 mmol) and 3-phenyl-1-propyne (0.065 mL, 0.522 mmol) in a mixture of water and *tert*-butyl alcohol (1:1, 1.1 mL) was added sodium ascorbate (0.025 mL, 1 M solution in water), followed by copper(II) sulfate pentahydrate (0.001 g, 0.004 mmol). The heterogeneous mixture was stirred at r.t. for 17 h, diluted with water, extracted with diethyl ether, dried (Na_2_SO_4_), filtered, concentrated and purified by chromatography (SiO_2_) eluting with hexane-ethyl acetate (1:1) to obtain the triazole **16** (0.086 g, 84%) as a colorless oil: IR (neat) 2953, 1713, 1611, 1514, 1446, 1245, 1174, 1046, 813, 725 cm^−1^; ^1^H-NMR (300 MHz, CDCl_3_) δ 7.33–7.19 (m, 7H), 7.09 (s, 1H), 6.90 (d, *J =* 8.7 Hz, 2H), 4.50–4.43 (m, 3H), 4.06 (bs, 2H), 3.82 (s, 3H), 3.68–3.58 (m, 1H), 2.56 (d, *J =* 7.5 Hz, 2H), 2.43–2.36 (m, 2H), 2.27–2.13 (m, 2H); ^13^C-NMR (75 MHz, CDCl_3_) δ 205.8, 159.7, 147.9, 139.3, 132.5, 128.9, 128.8, 127.3, 126.7, 122.1, 114.2, 78.6, 73.8, 55.5, 49.2, 47.4, 46.5, 36.6, 32.4; HRMS (ESI) *m/z* calcd for C_23_H_25_N_3_O_3_Na (M+Na) 414.1794, found 414.1768. 

*(2R,6R)-2-(4-Methoxyphenyl)-6-(2-(4-phenethyl-1H-1,2,3-triazol-1-yl)ethyl)dihydro-2H-pyran-4(3H)-one* (**17**). To a suspension of **3** (0.072 g, 0.262 mmol) and 4-phenyl-1-butyne (0.074 mL, 0.526 mmol) in a mixture of water and *tert*-butyl alcohol (1:1, 1.1 mL) was added sodium ascorbate (0.025 mL, 1 M solution in water), followed by copper(II) sulfate pentahydrate (0.001 g, 0.004 mmol). The heterogeneous mixture was stirred at r.t. for 14 h, diluted with water, extracted with diethyl ether, dried (Na_2_SO_4_), filtered, concentrated and purified by chromatography (SiO_2_) eluting with hexane-ethyl acetate (1:1) to obtain the triazole **17** (0.087 g, 82%) as a colorless oil: IR (neat) 2932, 1717, 1613, 1515, 1454, 1248, 1177, 1048, 831, 749 cm^−1^; ^1^H-NMR (300 MHz, CDCl_3_) δ 7.29–7.14 (m, 7H), 7.01 (s, 1H), 6.93 (d, *J =* 8.7 Hz, 2H), 4.48 (dd, *J* = 6.2, 7.2 Hz, 2H), 4.38 (dd, *J* = 5.9, 8.3 Hz, 1H), 3.83 (s, 3H), 3.52 (m, 1H), 3.09–2.93 (m, 4H), 2.57 (d, *J =* 5.7 Hz, 2H), 2.39–2.36 (m, 2H), 2.23–2.11 (m, 2H); ^13^C-NMR (100 MHz, CDCl_3_) δ 205.8, 159.7, 147.2, 141.3, 132.6, 128.7, 128.6, 127.3, 126.3, 121.7, 114.2, 78.4, 73.6, 55.5, 49.3, 47.4, 46.4, 36.6, 35.6, 27.5; HRMS (ESI) *m/z* calcd for C_24_H_27_N_3_O_3_Na (M+Na) 428.1950, found 428.1982. 

*(2RS,6RS)-2-(2-(1H-Benzo[d][1,2,3]triazol-1-yl)ethyl)-6-(4-methoxyphenyl)dihydro-2H-pyran-4(3H)-one* (**18**). To a solution of **3** (0.080 g, 0.291 mmol) in acetonitrile (1.5 mL) was added 18-crown-6 (0.173 g, 0.654 mmol) and cesium fluoride (0.121 g, 0.797 mmol). The reaction mixture was stirred at r.t. for 15 min and 2-(trimethylsilyl)phenyl trifluoromethanesulfonate (0.194 mL, 0.798 mmol) in acetonitrile (1 mL) was added. The reaction mixture was stirred at r.t. for 2 h, quenched with saturated sodium bicarbonate solution, extracted with diethyl ether, dried (Na_2_SO_4_), filtered, concentrated and purified by chromatography (SiO_2_) eluting with hexane-ethyl acetate (3:2) to obtain the triazole **18** (0.080 g, 78%) as a white solid: m.p. 137–138 °C; IR (neat) 2954, 1717, 1612, 1514, 1446, 1303, 1247, 1174, 1049, 1027, 813, 747 cm^−1^; ^1^H-NMR (300 MHz, CDCl_3_) δ 8.05 (dd, *J* = 1.8, 6.9 Hz, 1H), 7.44–7.27 (m, 3H), 7.31 (d, *J* = 8.7 Hz, 2H), 6.96 (d, *J =* 8.7 Hz, 2H), 4.89–4.77 (m, 2H), 4.43 (dd, *J* = 4.9, 9.4 Hz, 1H), 3.85 (s, 3H), 3.61–3.50 (m, 1H), 2.58–2.55 (m, 2H), 2.41–2.32 (m, 4H); ^13^C- NMR (100 MHz, CDCl_3_) δ 205.7, 159.7, 145.9, 133.5, 132.8, 127.5, 127.3, 124.1, 120.2, 114.3, 109.5, 78.7, 73.7, 55.5, 49.6, 47.5, 44.1, 36.5; MS (EI) *m/z* 374 (M+Na, 40%), 365 (100); HRMS (ESI) *m/z* calcd for C_20_H_21_N_3_O_3_Na (M+Na) 374.1481, found 374.1450. 

*(2RS,6RS)-2-(2-(1H-Pyrazol-1-yl)ethyl)-6-(4-methoxyphenyl)dihydro-2H-pyran-4(3H)-one* (**19**). To a solution of alcohol intermediate (0.091 g, 0.364 mmol), triethylamine (0.076 mL, 0.546 mmol) and DMAP (0.002 g, 0.016 mmol) in DCM (2 mL) was added methane sulfonyl chloride (0.034 mL, 0.437 mmol) dropwise at 0 °C. The resulting solution was stirred for 15 min, diluted with HCl (0.5 M) and extracted with DCM. The aqueous layer was back extracted with DCM, and the combined organic layers were washed with saturated sodium bicarbonate and brine. The organic layer was dried (Na_2_SO_4_), filtered and concentrated to obtain crude 2-((2*R*,6*R*)-6-(4-methoxyphenyl)-4-oxotetrahydro-2*H*-pyran-2-yl)ethyl methanesulfonate **2a **(0.120 g, quant.) as a yellow oil which was used without further purification. Pyrazole (0.029 g, 0.426 mmol) in DMF (1 mL) was added to a slurry of sodium hydride (0.013 g, 0.542 mmol) in DMF (3 mL) and stirred for 2 h. The resulting solution was added dropwise to a solution of the mesylate **2a** (0.120 g, 0.365 mmol) in DMF (0.5 mL) at 0 °C. The reaction mixture was stirred at 0 °C for 5 h, diluted with water, extracted with diethyl ether, dried (Na_2_SO_4_), filtered, concentrated, and purified by chromatography (SiO_2_) eluting with hexane-ethyl acetate (7:3) to afford **19** (0.022 g, 20% over two steps) as a colorless oil: IR (neat) 2956, 1713, 1514, 1247, 1027, 906, 725 cm^−1^; ^1^H-NMR (300 MHz, CDCl_3_) δ 7.52 (d, *J* = 1.5 Hz, 1H), 7.34–7.31 (m, 3H), 6.95 (d, *J =* 8.7 Hz, 2H), 6.22 (t, *J =* 1.8 Hz, 1H), 4.54 (dd, *J* = 5.4, 8.7 Hz, 1H), 4.34 (dd, *J* = 5.7, 7.8 Hz, 1H), 3.84 (s, 3H), 3.64–3.55 (m, 1H), 2.63–2.52 (m, 2H), 2.44–2.37 (m, 2H), 2.33–2.13 (m, 2H); ^13^C-NMR (100 MHz, CDCl_3_) δ 206.4, 159.7, 139.8, 132.9, 129.7, 127.2, 114.2, 105.5, 78.5, 74.1, 55.5, 49.5, 48.2, 47.6, 36.8; HRMS (ESI) *m/z* calcd for C_17_H_20_N_2_O_3_ 300.1474, found 300.1468.

*(2RS,4SR,6RS)-2-(4-Methoxyphenyl)-6-(2-(4-phenyl-1H-1,2,3-triazol-1-yl)ethyl)tetrahydro-2H-pyran-4-ol* (**20**). To a solution of **4** (0.040 g, 0.106 mmol) in MeOH (5 mL) at −10 °C was added NaBH_4_ (0.002 g, 0.053 mmol) in one portion. The reaction mixture was stirred at −10 °C for 4 h, quenched with water (2 drops), concentrated, purified by chromatography (SiO_2_) eluting with hexane-ethyl acetate (1:1) to afford **20 **(0.023 g, 72%-based on recovered **4**, 0.008 g) as an off-white solid: m.p. 139–140 °C; IR (neat) 3430, 2917, 1612, 1514, 1446, 1174, 1075, 1027, 701 cm^−1^; ^1^H-NMR (600 MHz, CDCl_3_) δ 7.78 (d, *J =* 7.2 Hz, 2H), 7.72 (s, 1H), 7.42 (t, *J =* 7.2 Hz, 2H), 7.33 (t, *J =* 7.2 Hz, 1H), 7.29 (d, *J =* 8.4 Hz, 2H), 6.89 (d, *J* = 8.4 Hz, 2H), 4.62–4.52 (m, 2H), 4.28 (dd, *J* = 1.8, 11.4 Hz, 1H), 3.96–3.91 (m, 1H), 3.82 (s, 3H), 3.45–3.42 (m, 1H), 2.28-2.23 (m, 1H), 2.19–2.13 (m, 2H), 2.02–1.99 (m, 1H), 1.88 (bs, 1H), 1.53 (ap q, *J* = 10.8 Hz, 1H), 1.35 (ap q, *J* = 11.0 Hz, 1H); ^13^C-NMR (100 MHz, CDCl_3_) δ 159.4, 147.7, 133.9, 130.9, 129.0, 128.3, 127.5, 125.9, 120.5, 114.1, 72.7, 68.3, 55.5, 47.1, 42.7, 40.9, 36.4; HRMS (ESI) *m/z* calcd for C_22_H_25_N_3_O_3_Na (M+Na) 402.1794, found 402.1766.

*(2RS,4RS,6RS)-2-(4-Methoxyphenyl)-6-(2-(4-phenyl-1H-1,2,3-triazol-1-yl)ethyl)tetrahydro-2H-pyran-4-ol* (**21**). To a solution of **4 **(0.050 g, 0.132 mmol) in THF (5.5 mL) at −90 °C was added L-Selectride (0.199 mL, 1 M in THF) dropwise over 10 min. The reaction mixture was stirred at −90 °C for 15 min, and quenched with saturated potassium sodium tartrate (10 mL). The mixture was diluted with diethyl ether (10 mL), stirred for 1 h, extracted with diethyl ether, concentrated, and purified by chromatography (SiO_2_) eluting with hexane-ethyl acetate (1:1) to give **21** (0.046 g, 92%) as an off-white solid: m.p. 127–128 °C; IR (neat) 3485, 2952, 1724, 1609, 1512, 1463, 1297, 1236, 1174, 1081, 1046, 826 cm^−1^; ^1^H-NMR (600 MHz, CDCl_3_) δ 7.76 (d, *J =* 7.2 Hz, 2H), 7.73 (s, 1H), 7.41 (t, *J =* 7.8 Hz, 2H), 7.34–7.31 (m, 1H), 7.29 (d, *J =* 8.4 Hz, 2H), 6.89 (d, *J* = 8.4 Hz, 2H), 4.80 (dd, *J* = 1.8, 12.0 Hz, 1H), 4.57–4.55 (m, 2H), 4.35–4.34 (m, 1H), 3.99–3.95 (m, 1H), 3.81 (s, 3H), 2.18 (bs, 1H), 2.21–2.15 (m, 1H), 2.11–2.05 (m, 1H), 1.91–1.89 (m, 1H), 1.78 (ddd, *J* = 2.6, 11.9, 14.2 Hz, 1H), 1.73 (dd, *J* = 2.3, 13.9 Hz, 1H), 1.62 (ddd, *J* = 3.0, 3.0, 12.0 Hz, 1H); ^13^C-NMR (100 MHz, CDCl_3_) δ 159.2, 147.7, 134.9, 130.9, 128.9, 128.2, 127.5, 125.9, 120.6, 114.0, 73.6, 69.3, 64.8, 55.5, 47.3, 40.4, 38.4, 36.6; HRMS (ESI) *m/z* calcd for C_22_H_25_N_3_O_3_Na (M+Na) 402.1794, found 402.1798.

## 4. Conclusions

In conclusion, a structurally diverse library of 2,6-disubstituted tetrahydropyrans featuring the DDQ-mediated C-H activation reaction has been developed. Most of the tetrahydropyrans were modified at the side chains via a click chemistry reaction to introduce substituted triazoles **4**-**17**. Several additional heterocyclic derivatives were also prepared. Diversity analysis showed that this library occupied unique chemical space compared to a 5 million “drug-like” compound collection as measured by the Tanimoto metric. The biological evaluation of this tetrahydropyran-based screening library is currently in progress. 
